# Percutaneous retrieval of migrated Viabahn stent from a segmental pulmonary artery

**DOI:** 10.1186/s42155-018-0007-3

**Published:** 2018-07-10

**Authors:** R. C. Zvavanjanja

**Affiliations:** Department of Diagnostic and Interventional Imaging, University of Texas Health Science Center at Houston, McGovern Medical School, 6431 Fannin St, MSB 2.132, Houston, TX 77030 USA

**Keywords:** Stent graft migration, Stent retrieval, Viabahn

## Abstract

**Background:**

With improving and aggressive endovascular venous and dialysis techniques there is increasing use of stent grafts with different platforms available on the market. Inappropriately sized stents may displace and potentially end up in the heart or lungs as potential dangerous foreign bodies. There is single published case of successful viabahn stent graft retrieval from the pulmonary circulation.

**Case presentation:**

We present a patient who had successful safe percutaneous retrieval of a migrated Viabahn stent from a segmental Pulmonary artery and describe a different novel safe technique of successful stent graft retrieval from the pulmonary artery with very low risk to potential damage to the cardiac valve complex.

**Conclusion:**

This case report demonstrates that Viabahn stent grafts can be safely retrieved from the pulmonary arterial system using this endovascular technique that will significantly reduce the risk of damage to the cardiac valve complex therefore avoiding potential complex surgery.

**Electronic supplementary material:**

The online version of this article (10.1186/s42155-018-0007-3) contains supplementary material, which is available to authorized users.

## Background

Over the last 10 years the role of stent grafts in dialysis access and venous interventions have significantly increased, this has been supported by a growing body of evidence supporting improved outcomes especially on venous outflow stenosis.

Given the variable venous sizes and often significant size transitions at venous anastomosis, stent undersizing may occur resulting in stent migration often terminating in the heart or pulmonary arterial circulation. There may be potential harm from the displaced stents in the pulmonary circulation and successful minimally invasive retrieval techniques may play a role in these situations.

There are previously described techniques of retrieval of bare metal stents from the pulmonary circulation in the past. One of the major concerns from these techniques has been potential damage to the valve complexes as the stents are removed endovascularly. There is also very limited literature describing successful retrieval of displaced stent grafts from the pulmonary circulation with a single case report describing Viabahn retrieval using a technique that was successful but could potentially damage the valve complex.

We present a case of Viabahn stent displacement with migration to the segmental pulmonary artery and subsequent successful safe retrieval using a relatively novel technique that significantly avoids damage to the valvular complex. To our knowledge this is the first time in published English literature that a stent graft, specifically Viabahn stent, that has migrated to the segmental pulmonary artery has been safely and successfully retrieved using endovascular technique.

## Case presentation

We present a 57 year old gentleman with CKD 5 who had an autogenous brachiocephalic fistula 4 months prior to presentation to us. During their last surgical clinical visit, the fistula was noted to be poorly maturing and then referred to interventional radiology for fistulogram and possible endovascular intervention to assist with fistula maturation.

The patient had a fistulogram which demonstrated a high grade juxta-anastomotic stenosis which was successfully balloon dilated. After a 6 week follow up clinic visit the fistula was still immature and a duplex scan, a second fistulogram with possible intervention were requested.

**Fig. 1:**
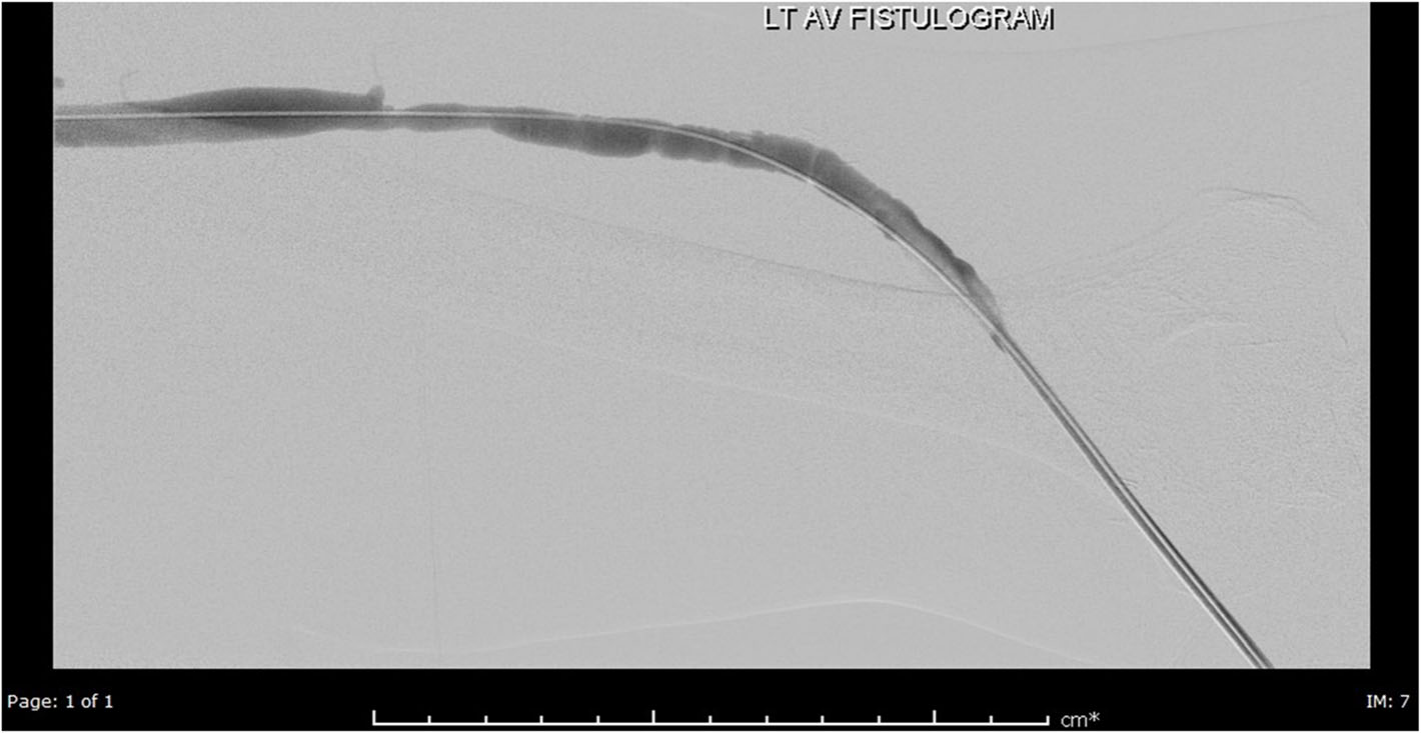
Post plasty fistulogram demonstrating “apparent recalcitrant” stenosis which predominantly spasm

**Fig. 2:**
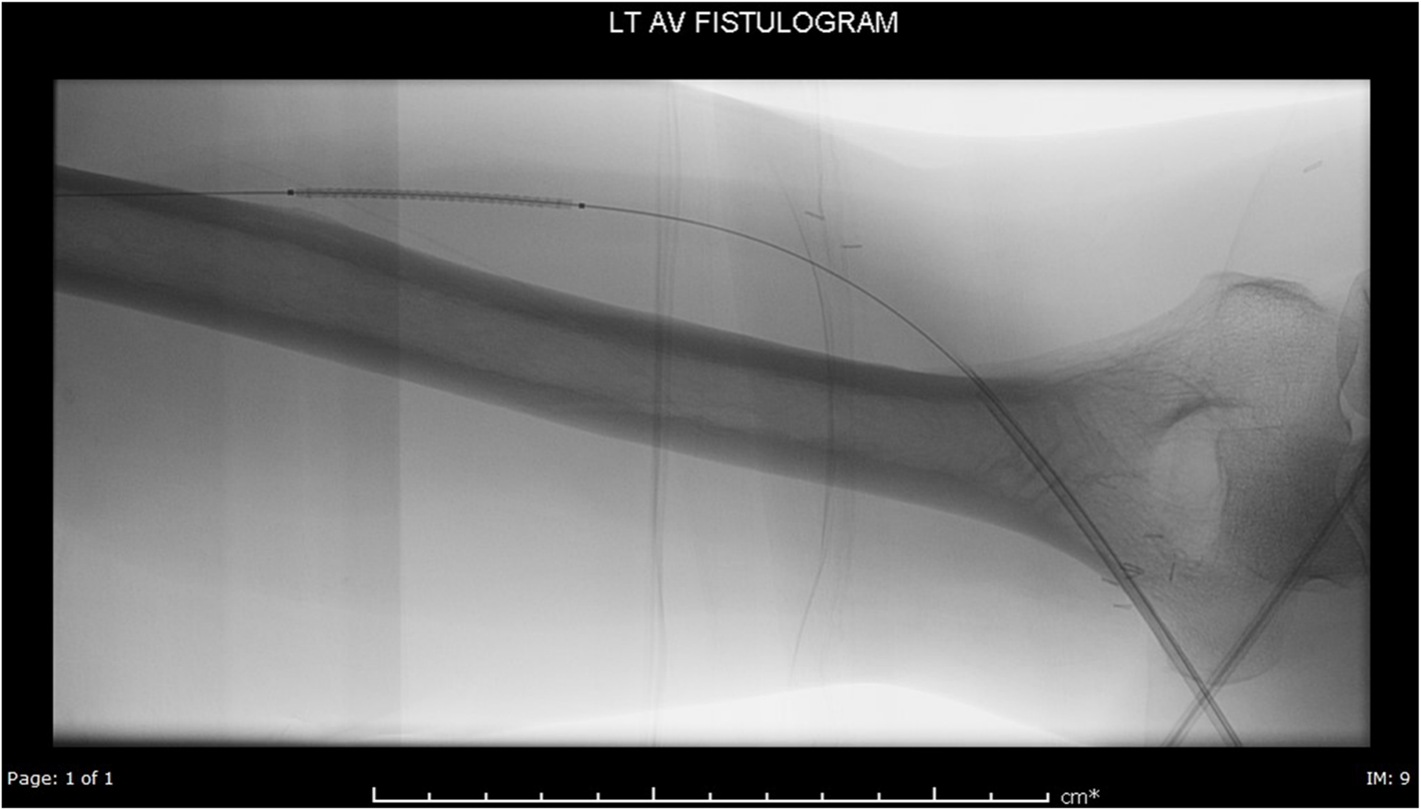
Pre stent (Viabahn 6 mm diameter) deployment images

Fistulogram was performed via an antegrade approach from an access just proximal to the swing point. There was an “apparent” stenosis (Fig. 1) which was angioplastied then followed by severe spasm (Fig. 2). which was perceived by the operator to be recalcitrant stenosis. In the light of this perceived recalcitrant stenosis, a decision to stent the area was taken. After measuring the vessel diameter based on the immediate post-plasty images a 6 mm diameter × 5 cm length Viabahn stent (Gore & Associates, Flagstaff, AZ) was selected and deployed in the standard fashion.

**Fig. 3:**
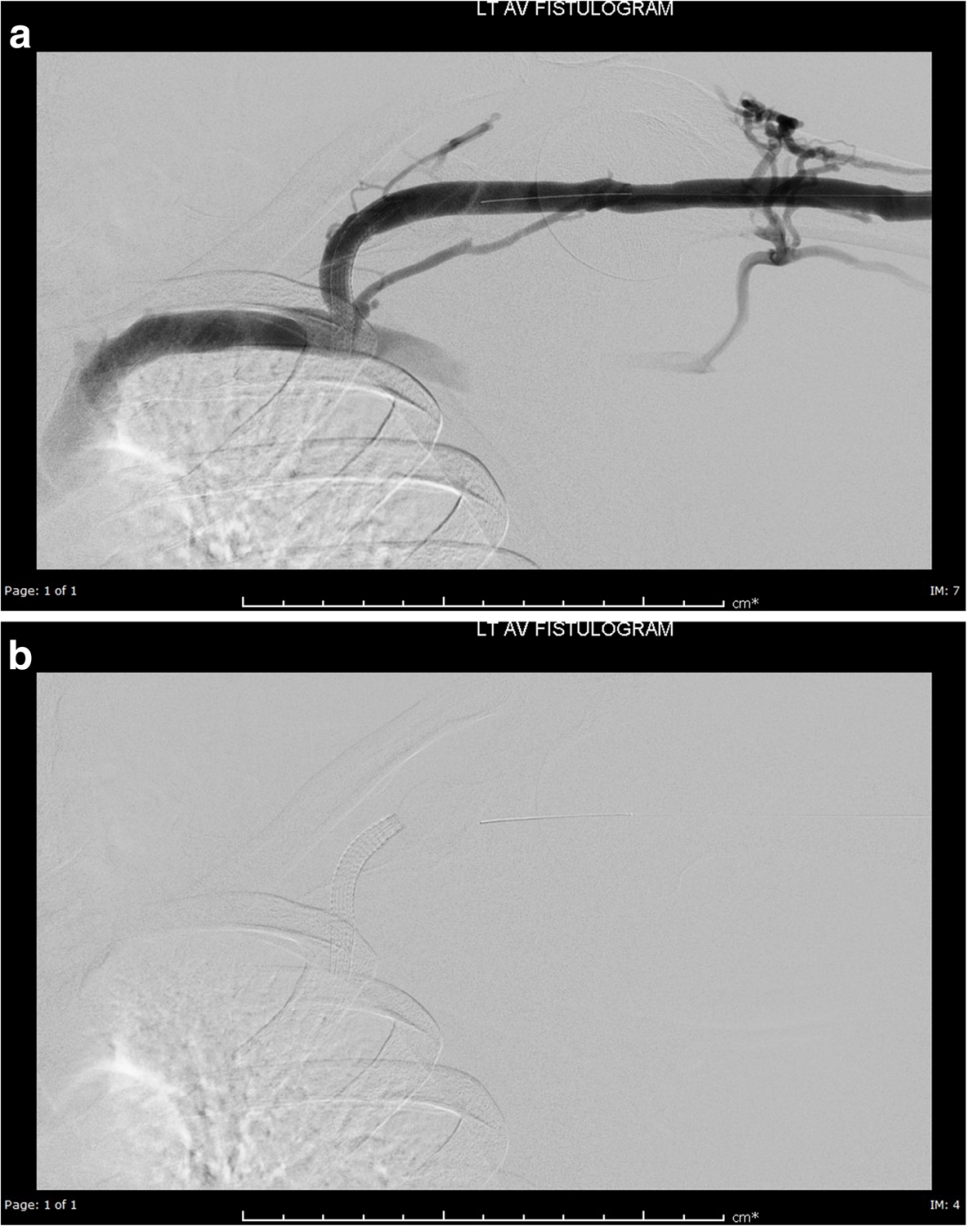
**a** and **b** Immediate post stent deployment fistulogram demonstrating stent migration to cephalic arch

Following stent deployment, the stent migrated and stopped at the confluence of the cephalic vein and the subclavian vein (Fig. 3). At the time the operator thought the stent was stable and unlikely to cause harm to the patient in this position. However, after reviewing the images with colleagues including vascular surgeons a decision was made to attempt to retract the stent into the arm which would be easier for the surgeon to retrieve the stent surgically, if required. The patient was subsequently brought back 24 h later to the interventional radiology suite.

**Fig. 4:**
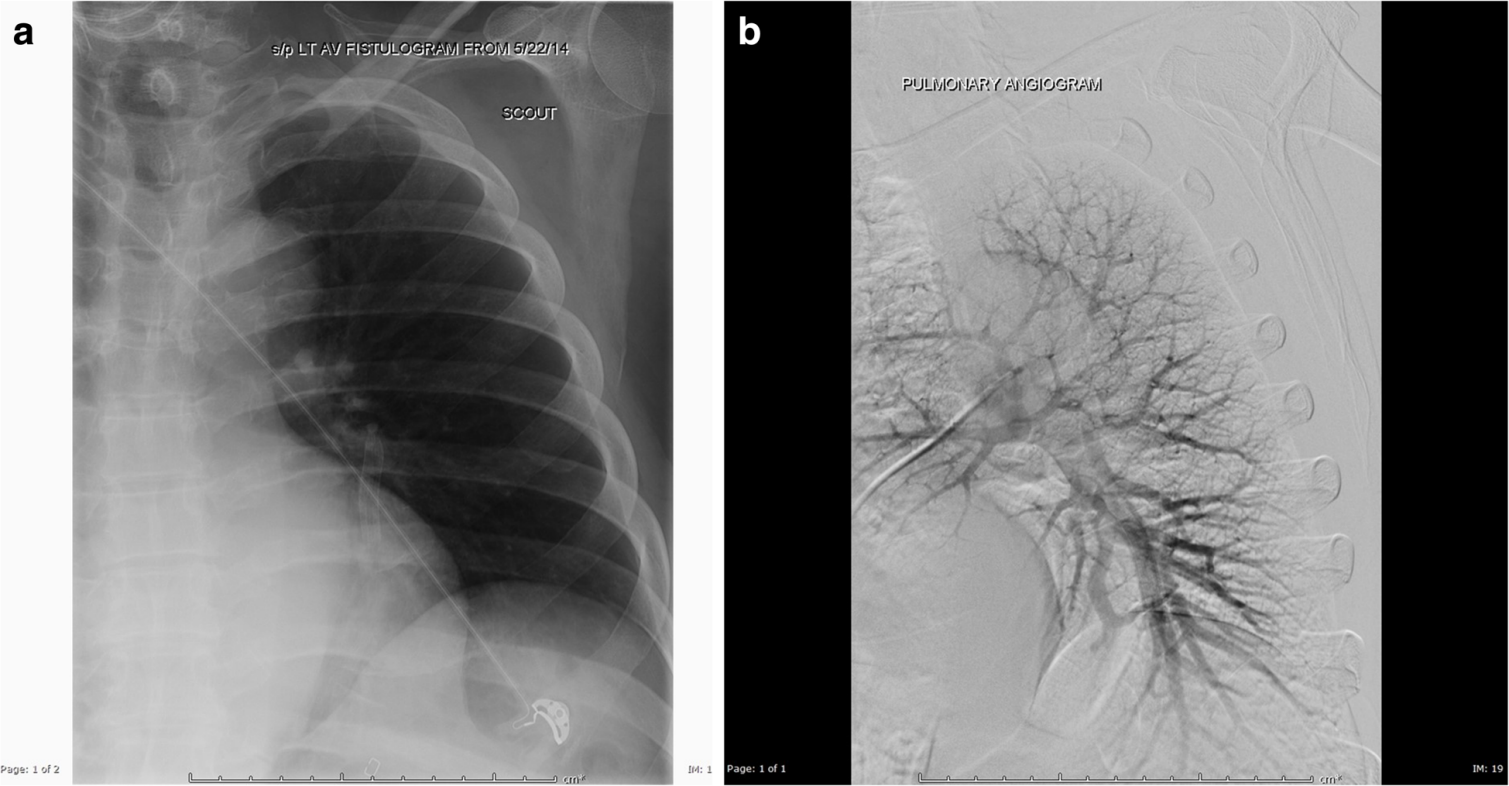
**a:** Images on the day the patient was brought in for stent retrieval. **b:** Pulmonary Angiogram demonstrating the stent in a segmental pulmonary arterial branch

Initial fluoroscopic image of the left shoulder region demonstrated the stent was absent from the final position documented the previous day indicating the stent had migrated further (Fig. 4a). Fluoroscopic scanning of the chest identified the stent to overlie the left lower lobe (Fig. 4a).

Subsequent Pulmonary angiogram confirmed the stent to lie within a segmental pulmonary artery of the left lower lobe (Fig. 4b).

After discussion of the options, risks and benefits with the patient and a multidisciplinary team, a decision to attempt stent retrieval was made versus leaving the stent in situ.

**Fig. 5:**
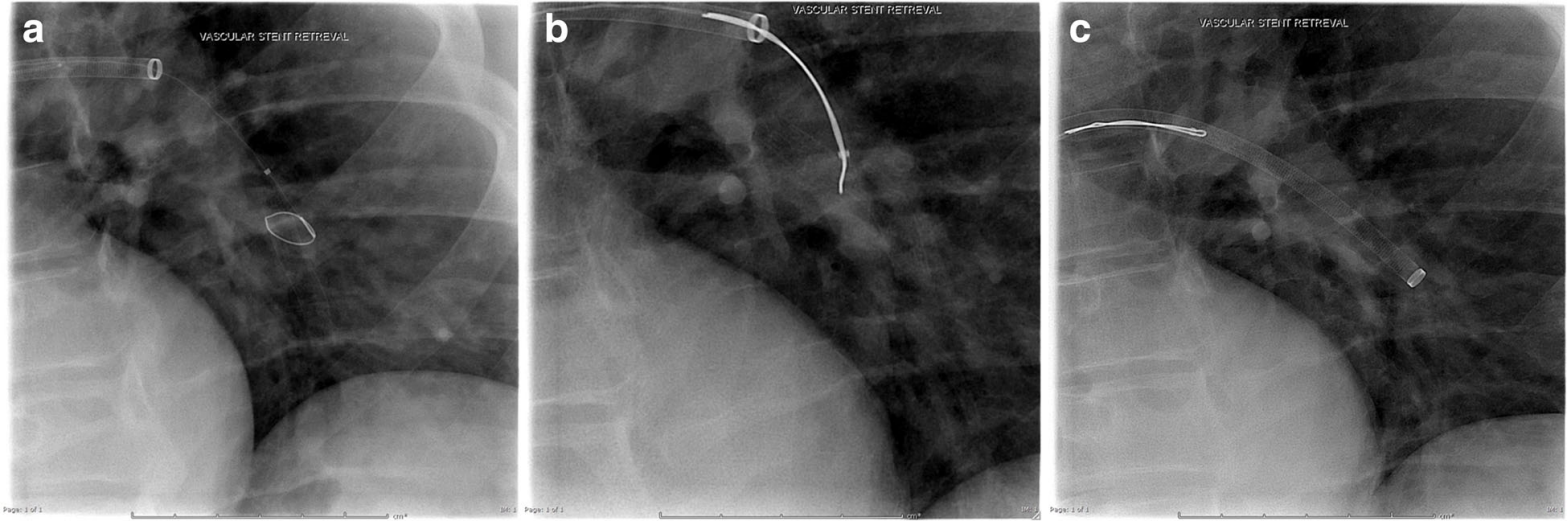
**a** and **b:** Demonstrates the Viabahn stent engaged around the midsection of the stent (**a**) and subsequently ensnared (**b**). **c:** The ensnared stent is “sheathed” over by the braided 12F sheath

**Fig. 6:**
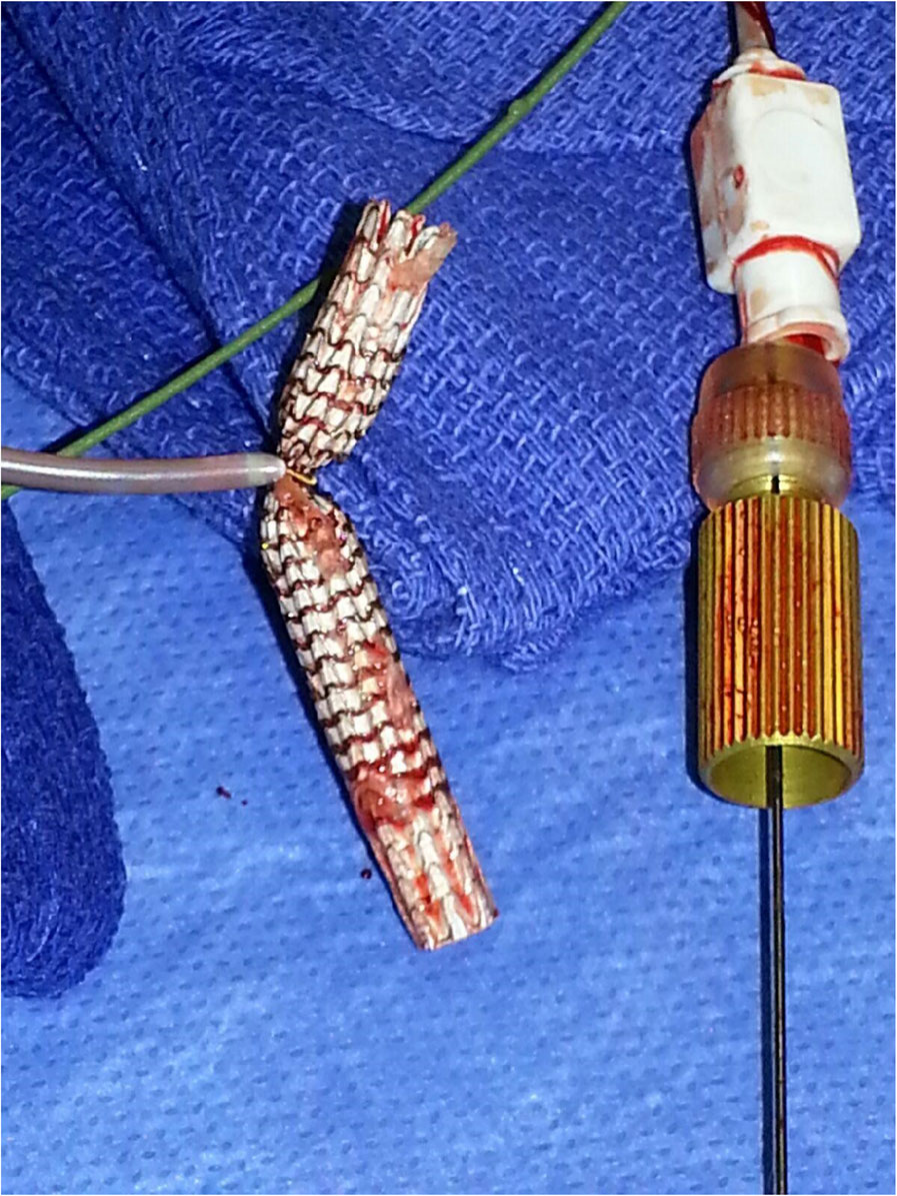
The snared stent outside the patient

After appropriate informed written consent, the right groin was prepped and in the standard fashion. Right common femoral vein access was then upsized to accept a 16 F sheath (Cook, Bloomington. IN USA). Main pulmonary access was then performed with an APC pulmonary catheter (Cook, Bloomington. IN USA). The APC catheter was then removed over a Storq wire (Cook, Bloomington. IN USA) wire and subsequently a 12 F 70 cm braided sheath was advanced into the main pulmonary artery and then left lower lobe pulmonary artery. Pulmonary angiograms performed identified the optimal projection to identify the vessel to access. After accessing the appropriate vessel the 12F sheath was advanced just to the origin of the branch above the stent. Subsequently a 15 mm Amplatz Gooseneck snare (ev3, Plymouth MN, USA) was manipulated until the stent was lassoed at about half way along the stent. Given the flexibility and potential collapsibility of the Viabahn stent it was over-sheathed carefully collapsing and gently retracting the captured stent to minimize potential vessel injury (Fig. 5a-c, Additional file 1). Once the stent had been totally ensheathed, the 12F sheath was retracted through the outer 16F sheath coaxially. The stent was retrieved intact (Fig. 6).

The procedure was performed under moderate sedation using Fentanyl and midazolam with continuous monitoring of the patient’s vitals by a dedicated nurse. Throughout the procedure the patient remained hemodynamically stable with normal respiratory function. Post procedure the patient was observed for 6 h post procedure before being discharged home in a stable condition.

The patient has so far been followed up for 3.5 years and has not developed any adverse pulmonary or cardiac condition. Interval CTPA done at an outside facility showed normal pulmonary vasculature with no evidence of pulmonary vessel injury.

## Discussion

To our knowledge this case is only the second case in published literature of successful percutaneous retrieval of a Viabahn stent that has migrated to the pulmonary arterial tree and indeed appears to be the first published case in which the migrated Viabahn stent graft has been retrieved from a segmental pulmonary artery.

In the only other published case by Dashkoff et al. (Dashkoff et al., 2010), there are similarities to our case: the Viabahn stents were lost during dialysis access work, the viabahn stents lost were both 6 mm diameter × 50 mm, in both cases minimally invasive percutaneous techniques were successfully used to retrieve the stents from the pulmonary vasculature and in both cases a decision to attempt retrieval was reached after a multi-disciplinary approach by all interested stakeholder clinical specialties. The later point cannot be over emphasized given the potential harm that could be incurred to a patient during pulmonary artery stent retrieval. In addition, there are mixed views in the literature with regards to whether foreign bodies in the pulmonary tree should be retrieved or adopt a “wait and see approach” (Gabelmann et al., 2001; Marcy et al., 2001).

There are however differences in technique between our case and Dashkoff et al.*’s* case which are worth discussing. Dashkoff et al., describe an interesting technique in which they snared the proximal end of the stent to create a cone after which they pulled the stent into the (Inferior vena cava) IVC passing through the right heart valve complex and then trapping the stent in a contralateral 16F sheath. This technique was effective however, the concern with retracting the exposed stent is always the potential to damage the valvular complexes. In contrast, our technique successfully sheathed the stent in the pulmonary artery and removed the sheath through the coaxially placed 16F sheath by so doing eliminating the risk of injuring the pulmonary and tricuspid valve complex. Unlike Dashkoff et al.*,* we were able to retrieve the stent with a single percutaneous access. Finally, we demonstrate that the viabahn can be safely retrieved endovascularly from the segmental pulmonary artery versus the lobar artery described by Dashkoff et al.

Our technique could potentially cause injury to the pulmonary artery but well synchronized sheath advancement and retraction of the snared stent under fluoroscopy guidance allows the operator to have a tactile and visual feedback of the situation thereby reducing the risk of vessel injury.

Other techniques that have been described previously with retrieval of bare metal stents in the pulmonary artery could have been used (Saeed et al., 1993; Ashar et al., 2002) in particular the balloon trapment technique and retraction of the complex/system has been widely described. The author opted against those techniques due to concerns over potential valvular complex injury (Slonim et al., 1999). Use of forceps to retract and reposition the stent has been described previously (Slonim et al., 1999; Berder et al., 1993) but was also decided against due to the probable high risk for pulmonary artery injury.

With the growing wealth of published data about the usefulness of the Viabahn stent grafts in dialysis access (Yevzlin & Asif, 2009) we are likely to see an increased utilization of these stent grafts in dialysis interventions and probably an increase in cases of migrated/mispositioned Viabahn stents, with some indeed reaching the pulmonary arteries. This case and that presented by Dashkoff et al.*,* demonstrate Viabahn stents can be safely retrieved from the pulmonary arteries with relative ease. The cases will probably increase the confidence of some interventionalists to attempt percutaneous retrieval of Viabahn stents if they migrate to the pulmonary tree in appropriate clinical situations.

## Conclusion

This case report demonstrates that Viabahn stent grafts can be safely retrieved from the pulmonary arterial system, particularly the segmental artery, using this endovascular technique that will significantly reduce the risk of damage to the cardiac valve complex therefore avoiding potential complex surgery.

## Additional file

Additional file 1: Video of the stent being “sheathed” over. (MP4 5314 kb)

## Author’s information

See front/cover page.
