# Correction to: Percutaneous retrieval of migrated Viabahn stent from a segmental pulmonary artery

**DOI:** 10.1186/s42155-019-0091-z

**Published:** 2020-01-06

**Authors:** R. C. Zvavanjanja

**Affiliations:** Department of Diagnostic and Interventional Imaging, University of Texas Health Science Center at Houston, McGovern Medical School, 6431 Fannin St, MSB 2.132, Houston, TX 77030 USA

**Correction to: CVIR Endovasc**

**https://doi.org/10.1186/s42155-018-0007-3**

In the published article (Zvavanjanja 2018) the statement under the subheading ‘Consent for publication’ is incorrect.

“Retrospective anonymized and no patient photos therefore no institutional requirement.”

should read:

“Informed consent for publication of this case report and its accompanying images has been obtained from the patient”
